# Use of modern contraception increases when more methods become available: analysis of evidence from 1982–2009

**DOI:** 10.9745/GHSP-D-13-00010

**Published:** 2013-07-26

**Authors:** John Ross, John Stover

**Affiliations:** a Futures Group International, Washington, DC, USA; b Futures Institute, Glastonbury, CT, USA

## Abstract

**Objective:**

To examine how much contraceptive use increases as additional methods are made available to populations.

**Methods:**

We used data estimating contraceptive use from representative national surveys and data estimating method availability from special surveys to make comparisons for 6 modern contraceptive methods, in each of 6 years: 1982, 1989, 1994, 1999, 2004, and 2009. To estimate method availability, we used various method accessibility rules governing different proportions of the total population (ranging from 20% to 80%) that must have access to a method in order for it to qualify as “available.”

**Results:**

Contraceptive use is greater when more methods are available to a large portion of the population, both cross-sectionally and over time. The addition of 1 method available to at least half the population correlates with an increase of 4–8 percentage points in total use of the 6 modern methods, for example, from 40% to 44% or 48%. A consistent pattern emerges for the relationship of contraceptive use and choice among multiple methods.

**Conclusions:**

Use of contraception may be increased by extending the availability of current methods, by improving features of current methods, or by introducing new methods. A wider choice of methods also improves the ability to meet the individual needs of women and couples. Although the method mix has been improving over time, current availability is far from ideal; in 2009, only about 3.5 methods, on average, were available to at least half of the population in the 113 countries included in our analysis.

## Abstract (precis)

International data over 27 years show that as each additional contraceptive method became available to most of the population, overall modern contraceptive use rose. But in 2009 only 3.5 methods, on average, were available to at least half the population in surveyed countries. Family planning programs should strive to provide widespread access to a range of methods.

## BACKGROUND

Use of modern contraception is prevalent across much of the developing world, but countries vary widely in total use and in the number and range of method choices available to potential users. For example, in sub-Saharan Africa the IUD is hardly available anywhere, but pills and injectables are generally available in the east and south. On the other hand, the IUD is commonly available in the Middle East. Female sterilization is not easily accessible in either region, but it is present in much of Latin America and in parts of Asia.^1^

No single method serves the needs of every subgroup in a population. The one-method programs established by some ministries of health exclude many people interested in using family planning and tend to result in low proportions of the population using contraception. In such cases, the addition of another modern method to a program's method mix can raise total use. Adding more methods helps up to a point, until diminishing returns set in. All this depends partly upon which methods are offered, but the very presence of more choices can assist users whose needs could not be met by any single method.

Analyses from 1971 onward have helped to demonstrate this relationship. For example, a Taiwan analysis showed a considerable rise in the duration of contraceptive use when family planning programs began offering multiple methods, estimating that the addition of one method would increase total contraceptive use by about 12 percentage points (from 30% to 42%).^2^ A simulation study demonstrated the limitations of any one method, noting that the usual discontinuation rate for a method would leave users with no alternative protection for their remaining reproductive years, except to have multiple abortions.^3^ A cross-national analysis found a systematic correlation between access to methods and their use.^4^ A recent study using detailed cluster-level data and service availability assessments from the Demographic and Health Surveys (DHS) demonstrated that improvements in family planning supply have a positive effect on contraceptive prevalence.^5^

Expanding the contraceptive method mix has been shown to improve continuation rates.

**Figure 1.**
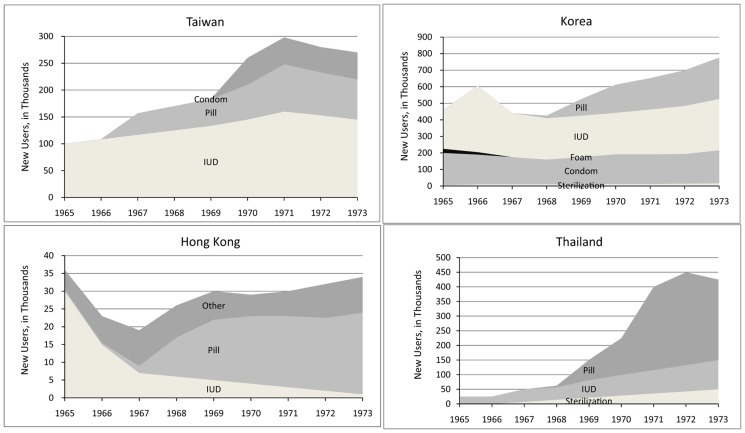
Impact of Adding New Contraceptive Methods on the Number of Users, 1965–1973 Reproduced with permission from Freedman R, Berelson B. The record of family planning programs. Stud Fam Plann. 1976, 7(1):1–40.

A striking example of the adoption of newly available contraceptive methods comes from the early experience of the first large-scale family planning programs in 4 Asian countries, during the advent of the IUD and pill (Figure 1). In these 4 countries, contraceptive use rose sharply after each new method emerged from the research stage and was made accessible to the population at large.^6^ Counter-examples, at least in part, include India and Pakistan, where the method mixes changed more erratically during the turbulent years of the late 1960s and 1970s.

The objective of this article is to extend previous research by examining how much contraceptive use increases as additional methods become available to whole populations, using national survey data and estimates of the degree of method availability.

## DATA SOURCES

Nationally representative data from the DHS series^7^ and from other national surveys compiled by the United Nations (UN) Population Division^8^ provide time trends for the modern contraceptive prevalence rate (MCPR), which includes use of male and female sterilization, IUDs, pills, injectables, implants, and condoms. We also refer to the contraceptive prevalence rate (CPR), which includes the previously mentioned modern methods, plus traditional methods, such as rhythm and withdrawal.

We obtained estimates of the MCPR for each country for each of the 6 years examined in our research from the surveys included in the UN compilation. Where surveys did not fall exactly on 1 of the 6 dates included in our analysis, we estimated the MCPR by interpolation between surveys bracketing the desired date. Where pairs of surveys were unavailable for interpolation, we set the MCPR value for the earliest years in question to the first available survey, and for years after the last available survey, we set the MCPR value to the latest available survey. This approach simplified the analysis and avoided questionable extrapolations before or after a survey estimate, although some error may be involved. The national surveys with MCPR data cover nearly every developing country, many with multiple surveys for various dates.

The number of contraceptive methods available in national family planning programs comes from the “Family Planning Effort (FPE) Index,” a score measuring the strength of national family planning programs on 4 dimensions (policies, services, evaluation, and method access) compiled through detailed questionnaires administered periodically over the past 4 decades.^1^ For the method access dimension, 10–15 expert observers from various professions and agencies in each country rate the extent to which the population has access to IUDs, pills, female sterilization, male sterilization, condoms, and, since 2004, injectables. (The addition of injectables to the questionnaire in 2004 could increase the number of available methods in our analysis for some countries, depending on the percentage judged to have access to the method.) Their responses are averaged after inspection for extreme outliers; as a further check, standard deviations are examined for each item in each country.

We used FPE Index data on method access from 1982, 1989, 1994, 1999, 2004, and 2009 to determine the number of methods available. Over all survey rounds, the FPE Index included 113 countries one or more times, drawn from all developing-country regions. In the survey rounds up to and including 1999, respondents rated “the percentage of couples of reproductive age who have ready and easy access to each method.” From 1999 to 2009, the FPE Index used a simpler questionnaire by asking respondents to rate the percentage of couples with access to each method on a scale from 1 to 10, which was then converted to the percentage estimate. (The 1999 index used both sets of ratings to compare the 2 methodologies.)

## METHODS

We considered a method to be “available” in the national family planning program if FPE Index survey respondents judged that a certain percentage (ranging from 20% to 80%) of the population had access to it. We used alternative accessibility rules of at least 20%, 40%, 50%, 60%, 70%, and 80% of the population having access to a method, in order to test the consistency of the relationships. These values were chosen somewhat arbitrarily but give a broad range and permit a check on the robustness of results when “availability” is defined differently. If we define method availability as low as a mere 20% of the population having access to the method, many more countries will be included in the analysis than if 60% of the population must have access to the method. The consistency of the conclusions under multiple assumptions is important since standard significance tests are not applicable, due to the complexities of sampling error in the original surveys compounded with survey errors in the access information for the 6 methods.

Table 1 shows how a more lenient accessibility rule increases the number of countries included in the analysis. It gives the number of countries with at least 1 available method qualifying under each accessibility rule, in each survey. The more severe the rule, the fewer the countries that qualify, as shown by the diminishing numbers in each column. In the 1982 survey, for example, 65 countries made 1 or more methods available, as defined by the lenient accessibility rule of only 20% of the population having access to the method, while only 26 countries met the stricter rule of 80% accessibility. An individual country therefore might have 4 methods, each accessible to 20% of the population, but only 1 method accessible to 80% of the population.

**Table 1.** Number of Countries With At Least 1 Available Contraceptive Method Included in the Analysis, by Method Accessibility Rule and Survey Year

Accessibility Rule^a^	Survey Year					
	1982	1989	1994	1999	2004	2009
20%	65	81	91	86	83	81
40%	46	68	84	85	83	80
50%	45	64	78	82	81	80
60%	38	57	71	79	77	80
70%	35	51	65	74	65	67
80%	26	40	59	53	41	39

a Per judgment of respondents to the Family Planning Effort Index survey that a certain percentage of the population in their country had access to a particular contraceptive method.

The other variable, the percentage of married/in-union women using modern methods (MCPR), is entirely separate, both empirically and conceptually. Accessibility/availability and use are quite different; a method may be widely available but little used. An example is the condom, which is generally available but used by only a small proportion of couples. (Another example is the traditional method of withdrawal, which is universally available but used only selectively.)

We conducted 4 different analyses using these data:

1. **Time trends:** to compare the trends in the mean MCPR and the mean number of available methods from 1982 through 2009
2. **Variability:** to compare variation in the MCPR with the average number of available methods, repeated for each year
3. **Correlational:** to examine the correlation across countries for the MCPR and the average number of available methods, repeated for each year
4. **Fixed effects regression:** to measure the relationship between method availability and MCPR, controlling for within-country variation

Although these 4 approaches explore the relationship of method availability and contraceptive use, they do not control for such confounding influences as the social setting, the health structure, or the particular method mix at each level of contraceptive use.

## RESULTS

Using these 4 approaches, we compare results for the relationship between contraceptive availability and use, selected from the 6 years and the 6 alternative contraceptive accessibility rules.

### Time Trends Analysis

**Figure 2.**
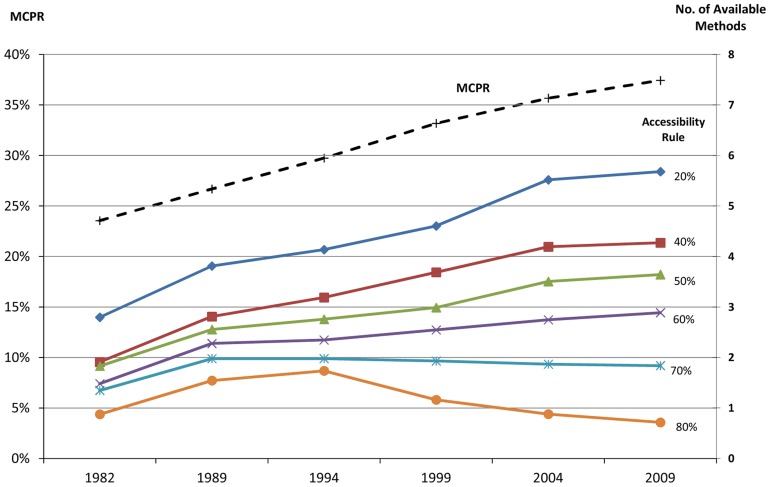
MCPR and Number of Available Methods, by Various Accessibility Rules, 1982–2009 Abbreviation: MCPR, modern contraceptive prevalence rate.

First, the number of methods available and the MCPR have both been increasing over time (Figure 2). The average MCPR rose from 23% to 37% between 1982 and 2009, represented by the dashed line in Figure 2. The solid lines show the average number of methods available among all surveyed countries in each year (right-hand axis), by the different accessibility rules. The more restrictive accessibility definitions (from 40% to 80%) produce lower estimates of the number of methods available. Nevertheless, each trend line for the accessibility rules from 20% through 60% rises in the analysis. The rise is more gradual (lower slope) as the accessibility percentage increases, and it is even negative at 80%. That is, the slopes rise less with more stringent levels of accessibility, probably because some saturation effect occurs once methods are fairly widely accessible.

Use of modern contraception, and the number of available methods from which people can choose, have both been increasing over time.

### Variability Analysis

**Figure 3.**
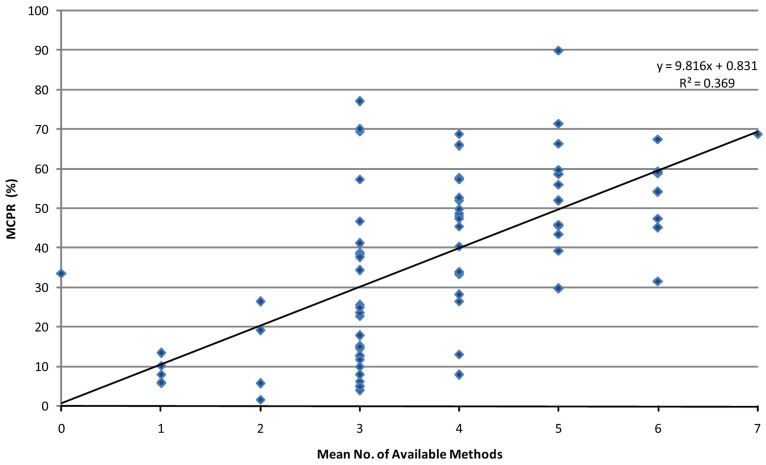
Relationship Between MCPR for 113 Surveyed Countries and Number of Available Methods, According to the 50% Accessibility Rule, 2009 Abbreviation: MCPR, modern contraceptive prevalence rate. Solid line represents the least squares line across all countries.

Second, cross-sectional correlations between the MCPR and the number of available methods for the 113 surveyed countries also show the MCPR-Availability correspondence. Figure 3 uses 2009 data to show the distribution of MCPR values for countries according to the average number of available methods according to the 50% accessibility rule. The least squares line gives an *R*^2^ value of 0.37 and a slope of 9.8, suggesting that 1 additional method raises the MCPR by nearly 10 percentage points in the latest survey round of FPE method accessibility scores.

The earlier survey years have somewhat smaller slopes of about 7 to 8 points (Table 2). Still, the patterns are again striking. Using the 50% accessibility rule for every year, once more the *R*^2^ values start high and descend (until 2009), while the slopes start low and rise.

Modern contraceptive use has increased over time with each additional method made available to the population.

**Table 2.** Relationship Between Mean MCPR and Mean Number of Available Methods According to the 50% Method Accessibility Rule, by Survey Year

Survey Year	*R*^2^	Slope
1982	0.66	7.2
1989	0.58	7.5
1994	0.43	7.4
1999	0.34	8.7
2004	0.22	8.0
2009	0.37	9.8

Abbreviation: MCPR, modern contraceptive prevalence rate.

There appears to be a large variation in the MCPR, especially at 3, 4, and 5 available methods (Figure 3), but this is partly because more countries fall into those categories, creating more data points. Nevertheless, the MCPR can vary due to a number of determinants that are not included in the analysis. For example, traditional contraceptive methods compete with and can reduce use of modern methods, as can extended breastfeeding. Also, where fertility rates are high, the proportion of women who are currently pregnant or postpartum is larger than in countries where fertility rates are lower, and total contraceptive use is lower. In addition, the uptake of available methods can be depressed where conservative attitudes prevail, as in some sub-Saharan African countries. Finally, the social setting matters; favorable socio-economic factors (such as education, income, urbanization) tend to raise contraceptive use levels, apart from the number of available methods. Much research over the years finds that the social setting and national family programs share credit in increasing contraceptive use levels: each has an independent effect, and both can act in concert.^9^

### Correlational Analysis

**Figure 4.**
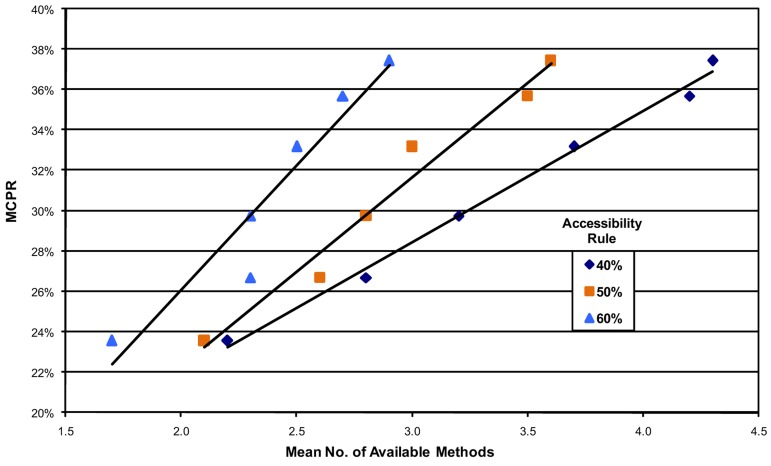
Relationship Between MCPR and the Number of Available Methods, by Accessibility Rule, 1982–2009 Abbreviation: MCPR, modern contraceptive prevalence rate. The 6 points on each line represent the 6 survey years, starting with 1982 at the lower left of each line and moving up to the right for 1989, 1994, 1999, 2004, and 2009.

Third, correlations show the rise in the MCPR with the increasing average number of available methods for each year. Figure 4 shows the relationships for the 40%, 50%, and 60% accessibility rules. The 6 points on each line represent the 6 survey years, from 1982 at the lower left to 2009 at the upper right, and each point shows the relationship between the average number of available methods and the average MCPR. For example, the 50% line in the middle starts low, with an average of slightly more than 2 methods available in 1982, rising regularly until in 2009, it exceeded 3.5 methods. Over the same time period, the average MCPR rose from just above 23% to about 37%. Regardless of the accessibility rule used, the association is always positive, and both values rise regularly over the years, in a linear pattern. (Note that the 40% line is positioned at the far right of the graph since it yielded the largest number of available methods.)

Regardless of the method accessibility rule used, modern contraceptive use always rises with each additional method made available.

We repeated this analysis for all accessibility rules, from 20% through 80% (Table 3). The *R*^2^ values start high (at the least stringent accessibility rule) and decrease, while the slopes start low and increase (until the strict 80% rule, for which fewer countries qualified). The essential point is that different accessibility rules, through 60%, show very consistent MCPR results, which fade only at the extremes of 70% and 80%. The increase in the MCPR ranges from 5–11 percentage points (indicated by the slope of the line) for each additional method made available. By the 50% accessibility rule, an increase of 1 new method is accompanied by nearly an 8 percentage point rise in the MCPR.

**Table 3.** Relationship Between Mean MCPR and Mean Number of Available Methods, by Method Accessibility Rule, 1982–2009

Accessibility Rule	*R*^2^	Slope
20%	0.97	4.9
40%	0.98	5.9
50%	0.96	7.9
60%	0.88	10.9
70%	0.25	11.2
80%	0.16	(5.3)

Abbreviation: MCPR, modern contraceptive prevalence rate.

### Fixed Effects Regression Analysis

Fourth, a “fixed effects” analysis measures the MCPR-Availability relationship with a control for within-country variation; this also corrects for the unequal numbers of data points among countries (some countries reported method access in all 6 survey years while others reported method access in fewer surveys; the average was 4.7 years of reporting).

**Figure 5.**
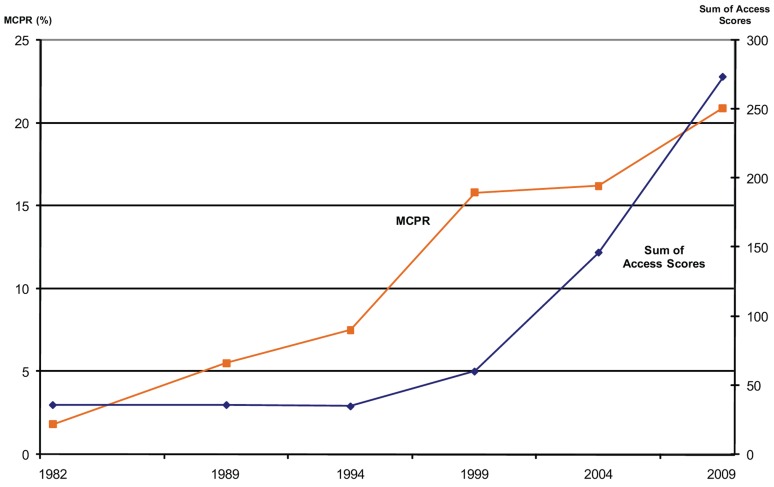
Relationship Between MCPR and Sum of Access Scores for All Modern Methods, Ethiopia, 1982–2009 Abbreviation: MCPR, modern contraceptive prevalence rate.

The fixed effects analysis finds the MCPR-Availability relationship within each country and then accounts for variations across the countries. As an example of the within-country relationship, Figure 5 uses the 6 surveys in Ethiopia. Over the 27 years from 1982 to 2009, availability improved steadily (indicated by the “sum of access scores” line), and the MCPR among married/in-union women rose from under 5% to 23%.

In this analysis, we summed the accessibility ratings for male and female sterilization, pills, IUDs, condoms, and injectables to create a total access score as a continuous variable. The resulting data set had 447 observations for 96 countries. The analysis, conducted with Stata 9.0, found that the access score is highly significant (*P*<.01, t_350_ = 11.9) and indicated an increase in MCPR of 4.5 percentage points (95% confidence interval = 3.8–5.3) with each additional method made available.

## DISCUSSION

Using multiple approaches to analyze the data, we found a consistently close correspondence between the number of available methods and the MCPR. The first 3 analyses showed that the addition of a new method raises the MCPR by approximately 7–8 percentage points, for example, from 40% to 47% or 48%, and somewhat more so in the latest survey data from 2009. The fixed effects regression in the fourth analysis confirms a significant relationship between method availability and MCPR, although the impact is at the low end of the range produced by the other approaches.

As noted above, our analyses cannot separate the effects of method availability on the MCPR from the ways by which increasing demand for contraception may encourage a program to make new methods available. But it does support the finding that greater availability of a number of contraceptive methods produces increases in MCPR over time.

The correspondence between method availability and the MCPR appears both over time (1982–2009) and cross-sectionally each year among the set of countries included in these analyses. This correspondence also exists under a variety of accessibility rules governing how much of the population must have access to a method for it to be regarded as an additional available method in the mix. (In the past, the 50% accessibility rule alone has been used to determine which methods, and which combination of methods, were available in most countries.^10^) And finally, the correspondence exists when the sum of contraceptive prevalence for all modern methods is the access measure.

As a country adds more methods to its offerings, it broadens the method mix, reducing “method skew,”^11^ which is a measure of the narrowness of the mix. A country that depends on only 1 or 2 prominent methods has more skew than a country with 3 or more methods. A broader offering gives options to more women and couples in the population with their differing needs. This tends to raise the MCPR.

A broader method mix helps meet the individual and varied family planning needs of women and couples.

The present results appear sufficiently strong to suggest that entirely new methods of family planning can increase modern contraceptive use in countries that make them widely available, giving more options to meet the needs of individuals. The impact may be less for new methods that are minor variations of existing methods, such as a new 6-month injectable compared with the existing 1- and 3-month injectables, although such new methods may improve contraceptive continuation rates.

In addition, a method that dramatically expands availability through lower cost or other advantages may be expected to have significant impact on contraceptive use, even if the method itself is not new. One example includes contraceptive implants, which are already growing in popularity and benefiting from markedly reduced pricing.^12^ Further, community-based distribution of some methods may be extended, for example by *Sayana® Press* (formerly known as depo-subQ provera 104™ in Uniject™)—a new subcutaneous formulation of *Depo-Provera®* packaged in the Uniject prefilled injection system.^13^

Introduction of new methods or improvements to features of existing methods may increase use of modern contraception.

The results of this analysis are not limited to inventions of new methods; they also suggest that increases in the MCPR may occur by simply widening geographic access to more of the existing methods. Patterns across 64 countries show that those with higher contraceptive prevalence tend to have a broader mix of methods.^1^ Currently, our analysis indicates that only about 3.5 methods are available, on average, to 50% of the population, and less than 2 methods are available by the 70% accessibility rule.

On average, only 3.5 methods are available to half of the population in surveyed countries.

This analysis also implies a significant effect of stockouts on contraceptive use. To the extent that stockouts of a method are equivalent to lack of availability as measured here, the effect on MCPR may be similar. One study showed an association between the CPR and the average availability of methods at service delivery points on the day of survey across 7 countries.^14^ The results show an effect roughly equivalent to a 5–6 point increase in CPR associated with availability of 1 additional method, not far from the 7–8 point increase emerging from 3 of our analyses.

There is the possibility of a dual influence between access to a method and popular interest in it. When it becomes clear that a method such as the injectable in eastern and southern Africa is taking off, programmatic efforts may be made to extend its availability to more clinics and to remote areas of the country. Conversely, if a new method is offered in selected areas but few women find it attractive or continue using it, efforts may slacken to supply it throughout the country or to seek budgetary funds to purchase large quantities of it.

### Limitations

Limitations to our analyses provide leads for further research. The results here do not control for possible confounding influences, such as education and income levels, the degree of urbanization, or differences in health systems. Similarly, programs that are intrinsically stronger may improve overall access in a variety of ways other than by adding to the number of available methods. These and other determinants of the MCPR and the CPR can be explored in future research, apart from the number of methods that are reasonably accessible to the population.

In addition, a more detailed focus on individual contraceptives would be of interest, to show the uptake of the pill alone, the IUD, or the injectable after each method becomes generally accessible in most of the country. Some of this research might be pursued by grouping methods into resupply and long-acting categories.

Which particular methods comprise the method mix as the mix changes over time is a topic of interest. A mix made up primarily of resupply methods may produce a lower MCPR than a mix that contains more long-acting methods, because of the inferior continuation rates of resupply methods compared with long-acting methods.

Within-country studies to compare differential responses to varying levels of accessibility by province would control some confounding variables that are present in cross-country research. Further, there are interesting questions about the factors that affect program managers' perceptions of which new methods promise widespread programmatic use, in light of their apparent advantages and disadvantages.

## CONCLUSION

Our research indicates that there is significant potential to increase contraceptive use by expanding access to existing methods and by making new or modified methods widely available. Although the method mix has been improving over time, as of 2009 only about 3.5 methods, on average, were available to half of the population in the 113 surveyed countries included here. Improving method availability would simultaneously expand benefits to individual women and to couples through wider contraceptive choice.
